# Comparative effectiveness of different probiotics combined with phototherapy for neonatal hyperbilirubinemia: a retrospective, non-randomized cohort study

**DOI:** 10.3389/fped.2026.1825590

**Published:** 2026-07-23

**Authors:** Xiangmiao Li, Yeyang Feng, Wanting Zhao, Feibing Liu, Zhen Cheng, Fengjie Sun

**Affiliations:** Department of Neonatology, The Fifth Affiliated Hospital of Guangzhou Medical University, Guangzhou, China

**Keywords:** bilirubin rebound, clinical efficacy, neonatal hyperbilirubinemia, phototherapy, probiotics

## Abstract

**Objective:**

This is a retrospective cohort study, not a clinical trial. No randomization, allocation concealment, or trial registration was performed. Phototherapy serves as the standard treatment for neonatal hyperbilirubinemia, yet clinical issues such as bilirubin rebound and prolonged hospital stay remain unresolved. This study compares the clinical efficacy and safety of two different probiotic regimens as adjuvant therapy to phototherapy.

**Methods:**

A retrospective analysis was conducted on 377 infants diagnosed with neonatal hyperbilirubinemia who were hospitalized in the Department of Neonatology, The Fifth Affiliated Hospital of Guangzhou Medical University between January 2021 and December 2023. Patients were divided into three groups according to the actual treatment received (phototherapy alone, phototherapy + Bifidobacterium quadruple viable tablets, or phototherapy + Bacillus subtilis and Enterococcus faecium viable granules). No randomization or blinding was applied due to the retrospective design. No trial registration was performed. Outcome measures included the dynamic trend of transcutaneous bilirubin, duration of phototherapy, length of hospital stay, total medical cost, and incidence of adverse reactions.

**Results:**

Both probiotic groups had shorter stays and fewer adverse reactions than phototherapy alone. After multivariable adjustment, Group B vs. A: hospital stay −0.52 days (95% CI: 0.24–0.80; *P* < 0.001); Group C vs. A: −0.29 days (95% CI: 0.01–0.56; *P* = 0.043); B vs. C: 0.24 days (95% CI: −0.04–0.51; *P* = 0.88). On day 3, estimated marginal mean transcutaneous bilirubin: B vs. A −2.83 mg/dL (95% CI: −3.63 to −2.03; *P* < 0.001); C vs. A −4.28 mg/dL (–5.07 to −3.50; *P* < 0.001); C vs. B −1.45 mg/dL (–2.24 to −0.67; *P* < 0.001). Bilirubin rebounded in Group A on day 3, while probiotic groups showed steady decline. Total serum bilirubin change and total cost did not differ among groups.

**Conclusions:**

Phototherapy combined with probiotics, particularly *Bacillus subtilis* and Enterococcus faecium viable granules, was associated with faster and sustained bilirubin reduction, helps prevent bilirubin rebound in the mid-treatment stage and shortens hospital stay, which may represent a potentially beneficial strategy for neonatal hyperbilirubinemia.

## 1 Introduction

Neonatal hyperbilirubinemia is a common disease in the neonatal period, affecting approximately 60% of full-term infants and 80% of premature infants (1). Its pathogenesis is mainly associated with the increased destruction of red blood cells during the neonatal period and the insufficient bilirubin metabolism capacity caused by immature liver function, which leads to the accumulation of bilirubin in the body. The clinical manifestation is jaundice of the skin. Without timely intervention, severe hyperbilirubinemia may lead to irreversible neurological sequelae, among which kernicterus is the most severe.

Phototherapy is currently the core therapeutic modality for this condition. Blue light with a specific wavelength (425–475 nm) converts unconjugated bilirubin into water-soluble isomers that can be excreted through body fluids, which effectively reduces serum bilirubin levels and prevents severe complications such as bilirubin encephalopathy (2). Although phototherapy has definite efficacy and is widely used, its clinical application still has several important limitations. In clinical practice, a subset of neonatal patients respond poorly to phototherapy, and some other patients experience transient bilirubin rebound after phototherapy discontinuation. These conditions often prolong hospital stays, increase medical burdens, and exacerbate maternal-infant separation anxiety. In addition, phototherapy may be accompanied by adverse reactions including fever, water-electrolyte imbalance, bronze baby syndrome, and retinal injury.

Recent studies have revealed that gut microbiota plays a critical role in the enterohepatic circulation of bilirubin. Microbe-derived β-glucuronidase is the core enzyme mediating this process, and its function has been validated in germ-free animal models. Furthermore, specific intestinal bacteria, including Clostridium perfringens and Bacteroides fragilis, can directly metabolize bilirubin. Brantley et al. (3) identified a putative bilirubin reductase BilR and confirmed its enzymatic activity, providing enzymological evidence that gut microbiota directly participates in bilirubin metabolism. Based on the aforementioned mechanisms, probiotics have been applied clinically as an adjuvant to phototherapy. Several studies have indicated that probiotics can reduce the duration of phototherapy for children with hyperbilirubinemia (4). In addition to jaundice, probiotics are increasingly utilized for the prevention of various neonatal conditions including feeding intolerance, necrotizing enterocolitis, bronchopulmonary dysplasia, and late-onset sepsis, with existing evidence supporting their safety and intestinal regulatory effects (5–8). Nevertheless, direct comparisons of the efficacy of different probiotic preparations as adjunctive therapy for neonatal hyperbilirubinemia remain limited at present. To address this critical research gap, the present study designed a large-sample retrospective cohort analysis based on neonates hospitalized with hyperbilirubinemia at a single tertiary care center. The primary objective is to compare the effectiveness of two widely clinically used probiotic preparations, as adjuncts to standard phototherapy, in accelerating the reduction of transcutaneous bilirubin levels and shortening length of hospital stay. The secondary objective is to evaluate the safety profiles and economic outcomes of each adjunctive treatment regimen. Accordingly, we propose the following research hypotheses: (1) compared with phototherapy alone, phototherapy combined with probiotics is associated with more rapid and sustained bilirubin reduction, a lower incidence of intermediate bilirubin rebound, and shorter hospital stay; (2) the magnitude of the aforementioned benefits differs between the two probiotic preparations, which reflects their distinct strain compositions and potential mechanisms of action. As this is a retrospective cohort study rather than a clinical trial, a single prespecified primary endpoint was not defined. Instead, the primary outcomes were prespecified as the dynamic trend of transcutaneous bilirubin and length of hospital stay (detailed in the Methods section). The findings of this study will clarify the comparative efficacy and cost-performance of different probiotic strains, and provide critical evidence for the formulation of clinical treatment strategies.

## 2 Materials and methods

### 2.1 Study design and participants

**Figure 1:**
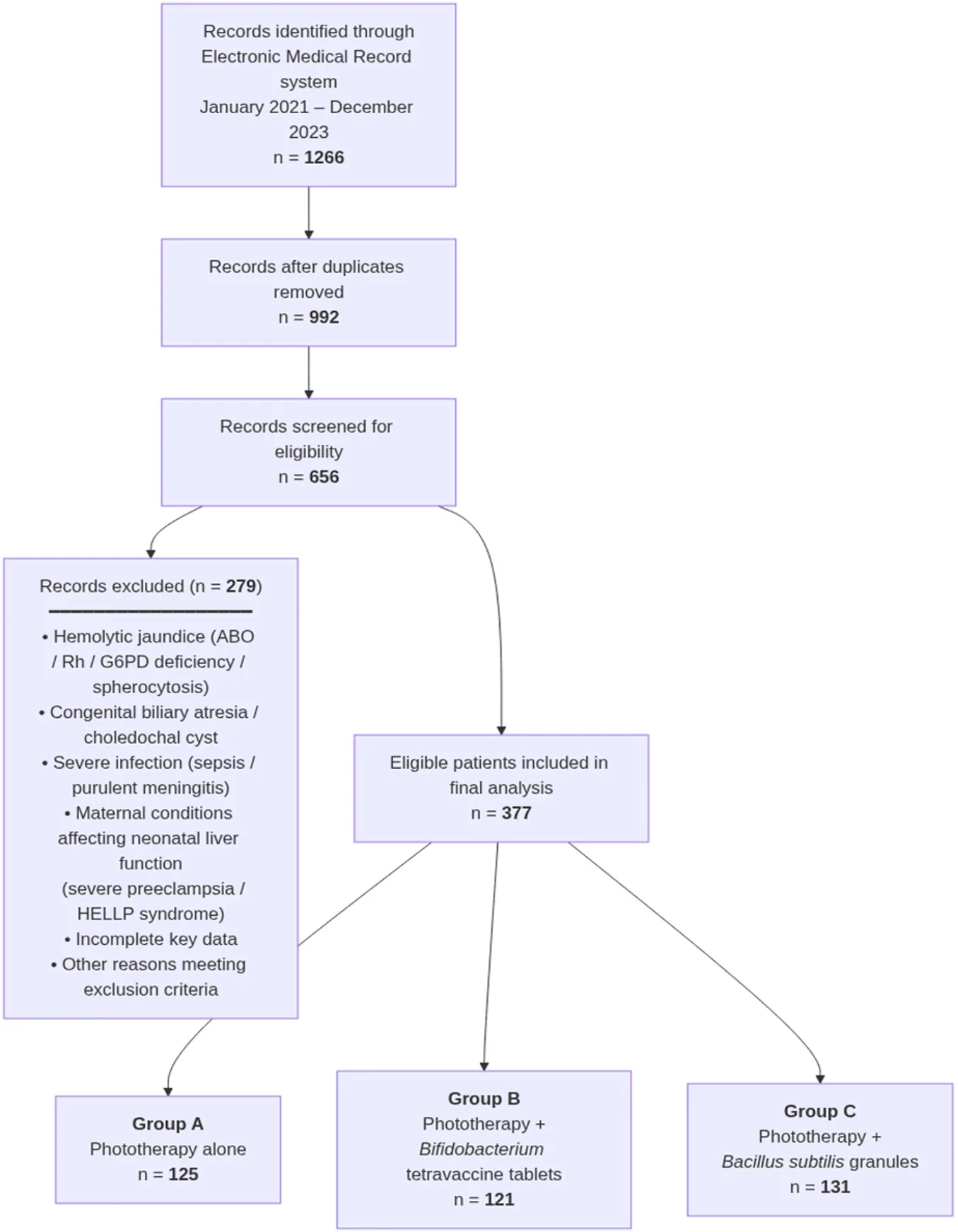
Flow diagram of participant selection.

This study adopts a retrospective, parallel-controlled cohort study design. We systematically screened hospitalized children admitted to the Neonatology Department of our hospital between January 2021 and December 2023. The enrollment procedure of study subjects followed strict screening criteria to ensure the homogeneity of the study population and the comparability of results. To accurately evaluate the effect of the intervention on idiopathic hyperbilirubinemia, this study established clear inclusion criteria based on hierarchical screening standards: (1) the patient is diagnosed with neonatal hyperbilirubinemia, and the total serum bilirubin level has reached the phototherapy intervention threshold corresponding to the patient's postnatal age and risk stratification (9) (2) gestational age ≥ 35 weeks and < 42 weeks (late preterm to term infants); (3) Apgar scores at 5 min and 10 min after birth are both >8 points; (4) vital signs are stable upon admission, with no critical conditions such as respiratory distress or circulatory instability. The exclusion criteria are as follows: (1) jaundice caused by specific etiologies including ABO or Rh hemolytic disease, G6PD deficiency, spherocytosis and other causes are excluded; (2) patients with end-stage organ diseases such as liver cirrhosis and renal failure that severely affect bilirubin metabolism and excretion are excluded; (3) patients with structural diseases of the hepatobiliary system such as congenital biliary atresia and choledochal cyst confirmed by imaging or laboratory examination are excluded; (4) patients complicated with severe infections such as sepsis and purulent meningitis are excluded, or those whose mothers have severe pregnancy complications that may affect neonatal liver function such as severe preeclampsia and HELLP syndrome are excluded; (5) patients with gestational age <35 weeks or ≥42 weeks are excluded. All patients meeting the inclusion criteria during the study period were enrolled. A *post-hoc* power analysis was performed to ensure that the observed difference in the primary outcome achieved a statistical power of at least 80%. A total of 1,266 neonatal medical records were retrieved from the hospital's electronic medical record system based on admission diagnosis and admission date. After removing duplicate cases and sequentially applying the preset inclusion and exclusion criteria, 889 cases were excluded, and a total of 377 eligible neonates with pathological jaundice were finally included in this study. The flow diagram of participant selection is presented in Figure 1 (Results section). All consecutive patients who met the inclusion criteria during the study period (January 2021 to December 2023) were enrolled. No selective sampling was applied. No sample size calculation or power analysis was performed before the study initiation, as this was a retrospective cohort study. The sample size (*n* = 377) was determined by the number of eligible consecutive patients during the study period.

### 2.2 Grouping and intervention protocols

Throughout the three-year study period (2021–2023), the phototherapy equipment, protocols, and the brands/batches of probiotics remained consistent. The phototherapy thresholds followed the same clinical guideline (Practical Neonatology, 4th edition).

All enrolled pediatric patients were divided into three groups based on the clinical treatment they actually received: Group A (phototherapy alone group, *n* = 125): patients received standard intermittent blue light therapy. No randomization procedure, sequence generation, or allocation concealment was applied, as the groups were determined by actual clinical treatment received. Blinding of participants, caregivers, clinicians, or outcome assessors was not performed due to the retrospective observational design. The assignment to probiotic combination groups was based on the attending neonatologist's clinical judgment and parental willingness, not randomization. Factors considered included the rate of bilirubin decline, expected phototherapy duration, and feeding tolerance. This non-randomized assignment introduces significant selection bias, which is a major limitation of this study.The specific protocol was as follows: a BL-60D blue light therapeutic instrument (Zhengzhou Disheng Instruments & Meters Co., Ltd.) was used, with a light source wavelength of 400–550 nm and irradiance stably maintained at 15–20 μW/cm^2^/nm. The specific treatment procedure was: the naked child was placed in a phototherapy box with servo temperature control, the distance between the light source and the body surface was fixed at 50 cm, and professional light-shielding eye masks and light-shielding diapers were used to tightly protect the eyes and perineum. The temperature of the phototherapy box was kept constant at 30–32 ℃, and the axillary temperature of the child was monitored hourly during the treatment to ensure that the core temperature was maintained within the physiological range of 36.5–37.2 ℃. The duration of phototherapy and intermittent frequency were dynamically adjusted according to the bilirubin level of the child. Phototherapy was initiated when total serum bilirubin reached the phototherapy threshold according to the 4th edition of Practical Neonatology. Interruption occurred when transcutaneous bilirubin fell 2 mg/dL below the threshold; phototherapy was resumed if rebound exceeded the threshold. Discontinuation was defined as two consecutive measurements (12 h apart) both below the safety threshold. Group B (combination of Bifidobacterium Tetravaccine Tablets and phototherapy group, *n* = 121): On the basis of the standard phototherapy administered in Group A, Bifidobacterium Tetravaccine Tablets were additionally given starting on the day of admission. Each tablet contains no less than 1.0 × 10^6^ CFU of Bifidobacterium infantis, no less than 1.0 × 10^6^ CFU of Lactobacillus acidophilus, no less than 1.0 × 10^6^ CFU of Enterococcus faecalis, and no less than 1.0 × 10^5^ CFU of Bacillus cereus. The administration method was: crush 0.5 tablet into powder, mix with approximately 5 mL of warm milk or warm water at 37 ℃ for oral administration, twice daily, continued until discharge. Group C (combination of Bacillus subtilis and Enterococcus faecium Granules and phototherapy group, *n* = 131): On the basis of the standard phototherapy administered in Group A, Bacillus subtilis and Enterococcus faecium Granules were additionally given starting on the day of admission. Each sachet (1.0 g) contains no less than 5.0 × 10^7^ CFU of Bacillus subtilis and no less than 5.0 × 10^7^ CFU of Enterococcus faecium. The administration method was: mix 0.5 sachet with approximately 5 mL of warm milk or warm water at 37 ℃ for oral administration, twice daily, continued until discharge. These two probiotic formulations were selected because they are the most commonly used compound probiotics for neonatal jaundice in Chinese clinical practice. The dosages followed the manufacturer's recommended pediatric doses. Their distinct strain compositions allow a comparative evaluation of potential mechanistic differences. Probiotics were initiated on the same day as phototherapy (the day of admission), with no delay between the two interventions. No concomitant treatments known to affect bilirubin metabolism (e.g., albumin, phenobarbital, exchange transfusion, intravenous immunoglobulin) were administered to any patient during the study period. The probiotic dosages followed the manufacturers' recommended pediatric doses. Treatment duration was from admission to discharge, as bilirubin levels had reached safe levels without rebound by the time of discharge, consistent with standard clinical practice (10). The use of adjuvant probiotics was determined by the attending neonatologist based on clinical judgment, with the main considerations including the rate of jaundice resolution, feeding tolerance, and parental willingness. Probiotics were preferentially administered to children with a slow initial decline in bilirubin or those expected to require a longer duration of phototherapy.

### 2.3 Observation indicators and data collection

Data were uniformly collected through the hospital electronic medical record (EMR) system to ensure the accuracy and completeness of information. The observation indicators were set as follows: (1) basic characteristics: gender, gestational age, birth weight, age on admission, and initial total serum bilirubin level; (2) efficacy and economic indicators: length of hospital stay, total phototherapy duration, difference in total serum bilirubin before and after treatment, transcutaneous bilirubin values on days 1–4 of treatment, and total hospitalization cost; (3) safety indicators: the incidence of adverse reactions such as rash, diarrhea, abdominal distension, fever, and bacteremia/sepsis was recorded. We then defined our primary outcomes as (1) the trend of transcutaneous bilirubin over the first 4 days, and (2) length of hospital stay. Secondary outcomes were phototherapy duration, total cost, and adverse reaction rates.In this study, bilirubin rebound during treatment was defined as an increase in transcutaneous bilirubin of ≥1.0 mg/dL compared with the previous measurement, which remained higher than the previous value, when bilirubin had shown a downward trend during continuous phototherapy and phototherapy was still ongoing or had just been discontinued. There were no missing data for the primary or secondary outcomes, as all enrolled patients completed the full course of treatment and daily bilirubin measurements were recorded according to hospital protocol.

### 2.4 Statistical analysis

Data were analyzed using IBM SPSS Statistics 25.0. The distribution of continuous variables was evaluated by the Shapiro–Wilk test and visually inspected with *Q*–*Q* plots. Data conforming to a normal distribution were expressed as mean ± standard deviation (SD) and analyzed using one-way analysis of variance (ANOVA) for intergroup comparisons; non-normally distributed data were analyzed using aligned rank transformation analysis of variance (ART-ANOVA). As a non-parametric method, ART-ANOVA does not require the assumption of a specific covariance structure for repeated measurements, unlike traditional repeated-measures ANOVA.Count data were expressed as number and percentage (%), and intergroup comparisons were performed using the chi-square test. Statistical significance was defined as *α* = 0.05.To adjust for confounders, we performed multivariable linear regression with length of hospital stay and day-3 TcB as outcomes, adjusting for gestational age, birth weight, age on admission, initial bilirubin, sex, and delivery mode (dummy-coded with Group A as reference). Variance inflation factor (VIF) was calculated, with VIF >5 indicating problematic collinearity. This study was not registered in a clinical trial registry because it is a retrospective cohort study. The study protocol is available from the corresponding author upon reasonable request.

## 3 Results

From January 2021 to December 2023, 1,266 records were identified from the electronic medical record system. After removing duplicates and applying the inclusion/exclusion criteria, 377 patients were finally included and divided into three groups based on actual treatment received.

### 3.1 Comparison of baseline characteristics

In this study, a total of 377 pediatric patients were enrolled, including 125 cases in Group A (75 males and 50 females), 121 cases in Group B (67 males and 54 females), and 131 cases in Group C (77 males and 54 females). No statistically significant differences were observed in baseline characteristics including gender distribution, gestational age, postnatal age, and birth weight among the three groups (*P* > 0.05), indicating that the three groups were comparable. For details, see Table 1.

**Table 1:** Demographics and baseline characteristics (*x̅* ± *s*).

Group	Gestational age (week)	Postnatal age (days)	Birth weight (kg)	Gender (male/female)	Mode of delivery (cesarean section/vaginal delivery)	Initial serum total bilirubin (μmol/L)
Group A (*n* = 125)	38.59 ± 1.25	4.39 ± 2.36	3.06 ± 0.42	75/50	28/97	287.53 ± 35.68
Group B (*n* = 121)	38.78 ± 1.13	4.63 ± 2.24	3.15 ± 0.35	77/54	31/90	277.43 ± 39.18
Group C (*n* = 131)	38.77 ± 1.19	5.06 ± 2.24	3.15 ± 0.35	77/54	32/99	286.25 ± 37.9
*P* value	0.088	0.052	0.97	0.748	0.836	0.074

### 3.2 Comparison of the differences in total serum bilirubin before and after treatment and transcutaneous bilirubin measurements on day 1 to day 4 after admission

**Figure 2:**
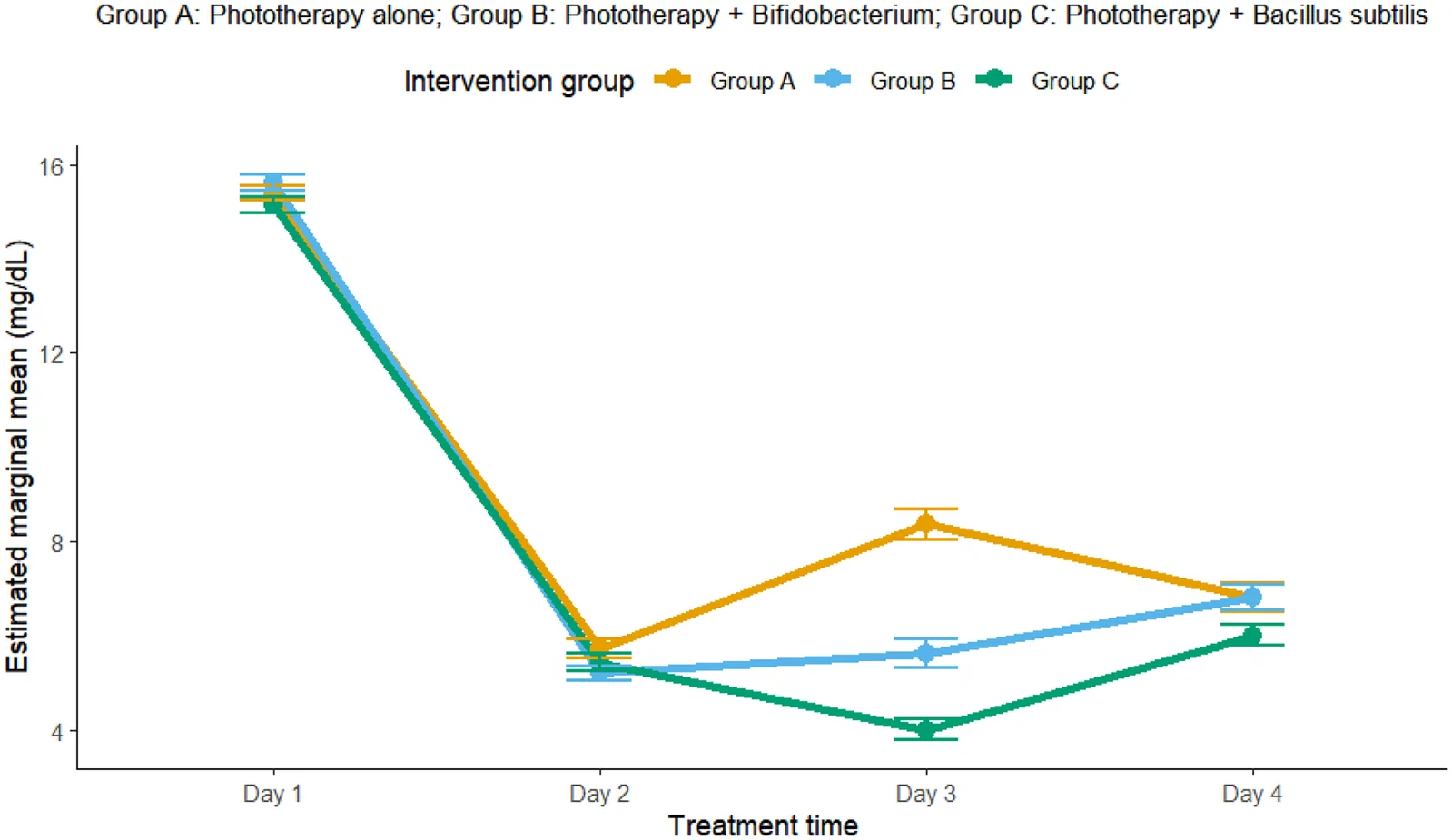
Trend of estimated marginal means for transcutaneous bilirubin in the three groups of infants.

**Figure 3:**
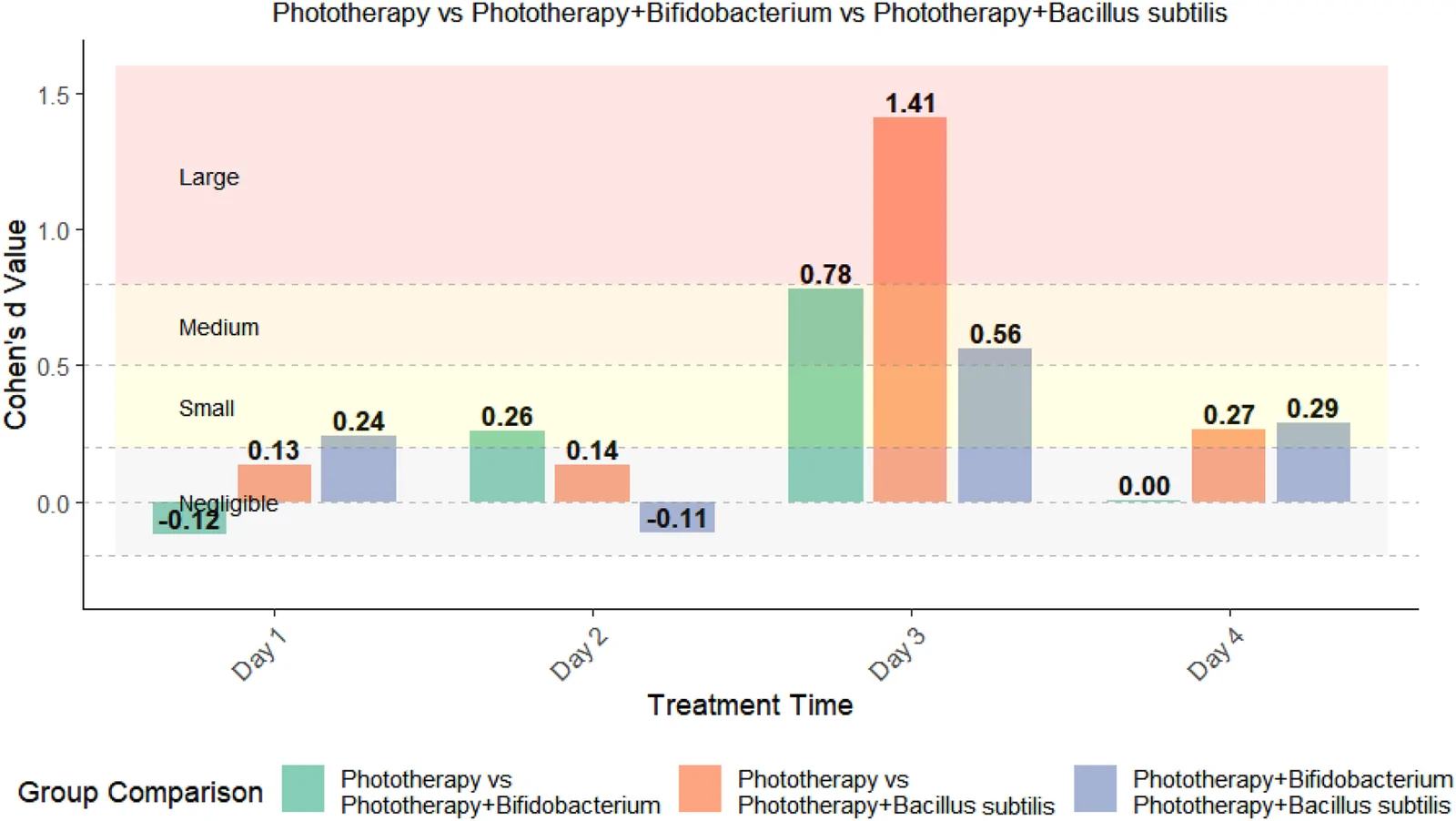
Cohen's *d* effect sizes for between-group comparisons at each time point.

Comparison of the difference in total serum bilirubin among the three groups of children before and after treatment showed no statistically significant difference (*P* > 0.05, Table 2). The interaction effect between time and group on transcutaneous bilirubin level was statistically significant (*P* < 0.001, Table 3). On the 3rd day after admission, the transcutaneous bilirubin level in group C was significantly lower than that in group B (estimated value = 1.6 mg/dL, *P* = 0.0198, Table 4). Figure 2 illustrates the trend of the estimated marginal mean of transcutaneous bilirubin over time across the three groups of children: the bilirubin level in group A peaked on day 3, while both group B and group C showed a continuous downward trend, with group C reaching the lowest level on day 3. The inter-group comparison results in Figure 3 show that on the 3rd day after admission, the bilirubin level in group A was significantly higher than that in the two combined treatment groups, and the difference between group A and group C was larger. The effect size results in Figure 3 indicate that on day 3, group C had a larger Cohen's *d* value compared with group A.

**Table 2:** Changes in Serum and transcutaneous bilirubin levels during treatment in the three groups (mean ± SD).

Group	Transcuaneous Bilirubin on Hospital Day 1 (mg/dL)	Transcuaneous Bilirubin on Hospital Day 2 (mg/dL)	Transcuaneous Bilirubin on Hospital Day3 (mg/dL)	Transcuaneous Bilirubin on Hospital Day 4 (mg/dL)	Changle in Serum Total Bilirubin (Before Vs After Treatment) (umol/L)
Group A (*n* = 125)	15.37 ± 1.74	5.74 ± 2.25	8.33 ± 3.67	6.83 ± 3.42	152.76 ± 47.36
Group B (*n* = 121)	15.60 ± 1.98	5.21 ± 1.74	5.63 ± 3.31	6.81 ± 2.81	149.51 ± 39.46
Group C (*n* = 131)	15.13 ± 1.94	5.43 ± 2.08	4.01 ± 2.41	6.02 ± 2.67	160.95 ± 38.86
*P* value	0.142	0.136	<0.001	0.059	0.086

1 mg/dL = 17.1 μmol/L.

**Table 3:** Results of aligned rank transform ANOVA for transcutaneous bilirubin values.

Source of variation	Degrees of freedom (df)	*F* value	*P* value	Partial *η*^2^ (*η_p_*^2^)	Effect size
Group	2	38.93	<0.001	0.17	Large
time	3	672.50	<0.001	0.64	Large
Group*Time interaction	6	20.08	<0.001	0.09	Medium

Partial *η*^2^ effect size interpretation: ηp² ≥ 0.14 indicates a large effect, 0.06 ≤ ηp² < 0.14 indicates a medium effect, and 0.01 ≤ ηp² < 0.06 indicates a small effect.

**Table 4:** Unadjusted pairwise comparisons for day-3 transcutaneous bilirubin: estimated marginal mean differences, Cohen's *d*, and *P* values .

Comparison groups	Mean Difference (mg/dL)	*P* Value	Cohen's *d*	95% Cl	Effect size
Group C VS Group A	−4.4	<0.0001	1.41	[1.13, 1.68]	Large
Group C VS Group B	−1.6	0.0198	0.56	[0.31, 0.82]	Medium
Group A VS Group B	2.7	<0.0001	0.78	[0.52, 1.04]	Medium

Interpretation of Cohen's *d* effect size: |*d*| = 0.2–0.5 indicates a small to medium effect, 0.5–0.8 indicates a medium to large effect, and >0.8 indicates a large effec; *P* values were adjusted using Bonferroni correction for three pairwise comparisons (*k* = 3).

No protocol deviations were observed. No patient crossed over between groups or discontinued the assigned probiotic treatment. All included patients completed the treatment as per their initial group assignment.

### 3.3 Comparison of length of hospital stay, duration of phototherapy and total hospitalization cost

There was a statistically significant difference in length of hospital stay among the three groups (*P* < 0.05). Specifically, the length of hospital stay in Group A was longer than that in the other two groups, while no statistically significant difference was observed between Group B and Group C (*P* > 0.05). No statistically significant differences were found in duration of phototherapy and total hospitalization cost among the three groups (all *P* > 0.05), as shown in Table 5.

**Table 5:** Comparison of length of hospital stay, phototherapy duration, and total hospitalization cost.

Group	Length of hospital stay (days)	Phototherapy duration (h)	Total hospital cost (yuan)
Group A (*n* = 125)	4.52 ± 1.25	57.41 ± 17.69	4,249.06 ± 1,105.07
Group B (*n* = 121)	3.92 ± 3.92	55.26 ± 16.26	4,232.35 ± 1,038.28
Group C (*n* = 131)	4.16 ± 1.10	59.03 ± 17.50	4,526.61 ± 907.52
*P* value	<0.001	0.22	0.09

### 3.4 Comparison of the incidence of adverse reactions between the Two groups during treatment

The difference in the incidence of adverse reactions among the three groups of children during treatment was statistically significant (*P* < 0.05). The incidence of adverse reactions in Group A was significantly higher than that in the two combination treatment groups. However, the difference in the incidence of adverse reactions between Group B and Group C was not statistically significant (*P* > 0.05), as detailed in Table 6.

**Table 6:** Comparison of the incidence of adverse events between the Two groups during treatment.

Group	Rash	Diarrhea	Fever	Abdominal distension	Bacteremia	Total, *n* (%)
Group A (*n* = 125)	23	1	0	0	0	24 (19.2%)
Group b (*n* = 121)	15	0	0	0	0	15 (12.3%)
Group c (*n* = 131)	11	0	0	0	0	11 (8.3%)
*P* value						0.037

No other adverse events were observed.

### 3.5 Multivariable regression analysis

To account for potential confounding, we performed multivariable linear regression adjusting for gestational age, birth weight, age on admission, initial total serum bilirubin, sex, and mode of delivery. The results are summarized in Table 7. After adjustment, both probiotic combination groups remained significantly superior to phototherapy alone in reducing day-3 TcB and shortening hospital stay (all *P* < 0.05). Group C (Bacillus subtilis and Enterococcus faecium granules) showed a significantly greater reduction in day-3 TcB compared with Group B (adjusted mean difference = −1.452 mg/dL, 95% CI: −2.239 to −0.665, *P* < 0.001). For length of hospital stay, Group B was significantly shorter than Group A (adjusted mean difference = −0.523 days, 95% CI: −0.804 to −0.243, *P* < 0.001), and Group C was also significantly shorter than Group A (adjusted mean difference = −0.285 days, 95% CI: −0.561 to −0.009, *P* = 0.043). There was no significant difference between Group C and Group B in hospital stay (adjusted mean difference = 0.239 days, 95% CI: −0.036 to 0.513, *P* = 0.88). No multicollinearity was detected (all VIF < 2.0).

**Table 7:** Multivariable linear regression analysis for length of hospital stay and day-3 transcutaneous bilirubin.

Outcomes Indicator	Comparison	AdjustedB	95% CL	*P*	VIF
Length of Hospital Stay (days)	B vs. A	−0.523	−0.804, −0.243	<0.001	1.389
	C vs. A	−0.285	−0.561, −0.009	0.043	1.399
	C vs. B	0.239	0.036,0.513	0.88	1.381
day-3 transcutaneous bilirubin (mg/dL)	B vs. A	−2.832	−3.631, −2.033	<0.001	1.374
	C vs. A	−4.284	−5.070, −3.499	<0.001	1.381
	C vs. B	−1.452	−2.239, −0.665	<0.001	1.387

Adjusted for gestational age, birth weight, age on admission, initial total serum bilirubin, sex, and mode of delivery. VIF, variance inflation factor. All VIFs were below 2.0, indicating no multicollinearity.

## 4 Discussion

Neonatal hyperbilirubinemia is a common disease during the neonatal period. Without timely intervention, it can lead to severe neurological sequelae such as bilirubin encephalopathy. Blue light phototherapy, characterized by simple operation, safety and effectiveness, is currently the first-line treatment for this disease. However, common adverse reactions such as diarrhea and fever may affect the treatment process. The occurrence of neonatal hyperbilirubinemia is mainly attributed to the immature development of neonatal liver function, which leads to relatively insufficient capacity for bilirubin clearance. In addition, the normal gut microbiota has not been established in the gastrointestinal tract. The high content and activity of β-glucuronidase derived from intestinal flora hydrolyze conjugated bilirubin into unconjugated bilirubin and glucuronic acid (11, 12). Unconjugated bilirubin is absorbed by intestinal cells, which promotes enterohepatic circulation of bilirubin. Meanwhile, due to weak intestinal peristalsis, bilirubin cannot be excreted with feces in time, which further affects enterohepatic circulation. Therefore, helping establish normal gut microbiota to promote bilirubin excretion has become a key target in clinical treatment. Based on this, probiotic preparations have been clinically used as a therapeutic modality for this disease. Bifidobacterium quadruple viable bacteria tablets and Bacillus subtilis double viable bacteria capsules are commonly used probiotic preparations in clinical practice. Several studies have confirmed that they can promote the establishment of intestinal flora and accelerate intestinal peristalsis, thereby accelerating bilirubin excretion (13–15).

The results of this study showed that the incidence of adverse reactions in the single phototherapy group was higher than that in the combination therapy group, and the difference was statistically significant (*P* < 0.05). A study by Tao et al. (16) involving 80 neonates with jaundice concluded that compared with the single phototherapy group, the incidence of fever, diarrhea and rash in the Bifidobacterium quadruple viable bacteria tablets combined with phototherapy group did not increase significantly. A study by Pan et al. (17) found that the combination of Bacillus subtilis double viable bacteria granules and intermittent phototherapy did not increase the incidence of adverse reactions. These findings collectively suggest that the combination of the probiotic preparations used in this study on the basis of phototherapy does not significantly increase additional safety risks, and the therapeutic safety is favorable. In addition, this study found that although there was no statistically significant difference in the decline of total serum bilirubin among the three groups before and after treatment, the length of hospital stay in the combination therapy groups was significantly shorter than that in the single phototherapy group (*P* < 0.05). Zhu et al. (15) divided 112 neonates with jaundice into the study group and the control group. The study group received phototherapy combined with Bifidobacterium quadruple viable bacteria treatment. The results showed that the study group had a shorter hospital stay than the control group receiving only phototherapy, and the levels of direct bilirubin, indirect bilirubin and total bilirubin in the study group were all lower than those in the control group. A study by Su et al. confirmed that blue light phototherapy combined with Bacillus subtilis double viable bacteria granules can reduce serum bilirubin, total bile acid and jaundice index levels, and shorten the jaundice resolution time (18).

More importantly, through dynamic monitoring of transcutaneous bilirubin levels during the treatment, this study found that phototherapy combined with probiotics (especially Bacillus subtilis double viable bacteria granules) can significantly accelerate the resolution of neonatal jaundice. This advantage is most prominent in the middle stage of treatment (the 3rd day), and can effectively stabilize the therapeutic effect and prevent the occurrence of rebound. The rebound observed in the single phototherapy group on the 3rd day reveals the instability of its efficacy, which may be related to the increased reabsorption via enterohepatic circulation after the cessation of phototherapy. Through colonization and metabolic effects in the intestine, the two probiotic regimens, especially Bacillus subtilis double viable bacteria granules, may effectively block the enterohepatic circulation of bilirubin and promote its excretion with feces, thereby achieving a more stable and sustained decreasing trend. Furthermore, in the direct comparison of the two probiotics, Bacillus subtilis double viable bacteria granules showed a more favorable association compared with Bifidobacterium quadruple viable bacteria tablets. After adjustment for confounders, the mean difference on day 3 was −1.45 mg/dL (95% CI: −2.24 to −0.67, *P* < 0.001).This is a useful complement to the study by Tao Lijun et al, which confirmed that Bacillus subtilis double viable bacteria enteric-coated capsules are superior to Bifidobacterium triple viable bacteria capsules in terms of both effectiveness and cost-effect ratio for adjuvant treatment of neonatal jaundice, but did not deeply reveal the dynamic change trend of bilirubin during treatment (19). A study on healthy neonates showed that early postpartum supplementation with probiotic combination containing Lactobacillus and Bifidobacterium can significantly reduce transcutaneous bilirubin levels, delay the initiation of phototherapy and shorten the length of hospital stay, and the formula containing Bifidobacterium has the most prominent effect in promoting bilirubin elimination (20). When combined with intravenous immunoglobulin and phototherapy, Bifidobacterium triple viable bacteria is superior to Bacillus subtilis double viable bacteria or Saccharomyces boulardii in reducing total serum bilirubin, improving jaundice resolution time and milk intake (21). These two findings do not contradict the dynamic advantage of Bacillus subtilis double viable bacteria granules observed in this study; instead, they indirectly indicate that the adjuvant efficacy of probiotics is highly dependent on the disease type and combined intervention measures.

In this study, the difference in the efficacy of the two probiotic preparations may be attributed to the differences in their modes of action and strain characteristics. First, the combined Bacillus subtilis and Enterococcus faecium granules rapidly consume oxygen in the intestinal tract via the included aerobic bacterium (Bacillus subtilis) to create an anaerobic environment. This not only facilitates the colonization of its associated bacterium (Enterococcus faecium), but also provides favorable conditions for the proliferation of the host's endogenous obligate anaerobes (22). In contrast, the combined Bifidobacterium quadruple viable bacteria tablet directly replenishes exogenous flora, such as Bifidobacterium infantis and Lactobacillus acidophilus. Although the Bacillus cereus contained therein can consume oxygen temporarily, it cannot achieve long-term colonization (23), rendering the preparation more dependent on the colonization success rate of the exogenous flora. Second, Bacillus subtilis exists in the form of spores, which exhibits high tolerance to gastric acid and bile, ensuring that a large number of viable bacteria can reach the colon; while vegetative forms such as Bifidobacterium are more sensitive to the gastrointestinal environment, and may suffer greater loss during the delivery process. Furthermore, Bacillus subtilis can also produce antimicrobial peptides to directly inhibit pathogenic bacteria (24). Relevant studies indicate that specific strains of Bacillus subtilis may affect the 5-hydroxytryptamine signaling pathway by regulating intestinal tryptophan metabolism, thereby enhancing gastrointestinal motility (25). These mechanisms collectively underpin the rapid and steady onset of action of Bacillus subtilis preparations. In comparison, the combined Bifidobacterium quadruple viable bacteria tablet relies more on the fermentation and acid production after colonization and the inhibition of *β*-glucuronidase activity to reduce the enterohepatic circulation of bilirubin, which is a relatively slow process.

This study conducted a direct comparison of two commonly used probiotic preparations, providing practical evidence for clinical decision-making. In addition, this study adopted ART-ANOVA to conduct robust repeated measurement analysis on the change trajectory of bilirubin, and revealed the rebound phenomenon in the mid-treatment period in the simple phototherapy group, which has received limited attention in previous studies. Meanwhile, the sample size of this study is relatively large (*n* = 377), providing sufficient statistical power for detecting inter-group differences with clinical significance.

This study has several limitations. Because treatment assignment was based on clinician judgment and parental willingness rather than randomization, selection bias is unavoidable, so our findings should be interpreted as associations, not causal effects. The retrospective design also carries risks of unmeasured confounding, though baseline characteristics were balanced; moreover, key clinical variables—feeding method, weight loss percentage, dehydration status, and maternal factors—were not available from the medical records and could not be analyzed, meaning these unmeasured confounders may have influenced the results. The single-center design further limits generalizability, and we did not monitor probiotic compliance, so we cannot be sure that all patients received the full prescribed dose.

We did not measure gut microbiota or β-glucuronidase activity, leaving our mechanistic explanation inferential, and our comparison of the two probiotic regimens may not apply to other strains or dosages. Follow-up was short, and long-term neurodevelopmental outcomes were not assessed; no sample size or power analysis was performed before the study, which may limit detection of small effects. Finally, due to the retrospective design, the evidence level of this study is lower than that of randomized controlled trials.

We should also note that our definition of bilirubin rebound (a rise of ≥1.0 mg/dL in TcB) was chosen to track day-to-day changes during phototherapy. There is no single international standard for defining rebound—definitions vary by study purpose. Our definition is not arbitrary; it fits our aim of monitoring real-time fluctuations. However, it differs from the more clinically oriented definition used in some prediction studies (return to phototherapy threshold within 72 h after stopping phototherapy), so readers should keep this difference in mind when interpreting our rebound findings.

In terms of safety, no probiotic-related bacteremia, sepsis or serious adverse events were recorded in either group. The observed adverse events were mainly mild rashes, all of which resolved spontaneously without requiring special intervention. However, limited by retrospective data collection, mild and transient gastrointestinal symptoms, which are often associated with probiotic use but have not been systematically recorded, may be underestimated. Given that the immune function of newborns is not yet mature, clinicians should pay attention to the rare but potential risk of probiotic-related sepsis, especially for preterm infants or children with impaired immune function.

## 5 Conclusion

Based on the findings of this study, the combination of probiotic adjuvant therapy on the basis of conventional phototherapy was associated with consolidated therapeutic efficacy, reduce mid-term rebound rate and shorten the length of hospital stay. A comparison between the two compound probiotic preparations shows that the live combined Bacillus subtilis and Enterococcus faecium granules showed a more favorable outcome the live combined Bifidobacterium, Lactobacillus, Enterococcus and Bacillus cereus tablets in promoting early bilirubin clearance and preventing mid-term rebound. However, limited by the single-center retrospective design and sample size of this study, the above conclusions still require further verification through large-scale prospective studies.

## Data Availability

The raw data supporting the conclusions of this article will be made available by the authors, without undue reservation.
