# Correction: Comparative effectiveness of different probiotics combined with phototherapy for neonatal hyperbilirubinemia: a retrospective, non-randomized cohort study

**DOI:** 10.3389/fped.2026.2016511

**Published:** 2026-10-19

**Authors:** Xiangmiao Li, Yeyang Feng, Wanting Zhao, Feibing Liu, Zhen Cheng, Fengjie Sun

**Affiliations:** Department of Neonatology, The Fifth Affiliated Hospital of Guangzhou Medical University, Guangzhou, China

**Keywords:** bilirubin rebound, clinical efficacy, neonatal hyperbilirubinemia, phototherapy, probiotics

The Equal contribution statement for authors Xiangmiao Li and Yeyang Feng was erroneously ommitted.

The original version of this article has been updated.

