# Supplementary material for: Comparative effectiveness of different probiotics combined with phototherapy for neonatal hyperbilirubinemia: a retrospective, non-randomized cohort study
Source: Front Pediatr. 2026 Jul 23;14:1825590. doi: 10.3389/fped.2026.1825590 (PMC13445554; doi:10.3389/fped.2026.1825590)
Supplement: Supplementary file 1 [file Table1.docx]

Table S1.Results of normality and homogeneity of variance tests for transcutaneous bilirubin (TcB) across groups and time points.

| Time point | Group | Shapiro-Wilk | | Levene's test | |
| --- | --- | --- | --- | --- | --- |
|  |  | W | *P* | *F* | *P* |
| Day 1 | A (n=125) | 0.985 | 0.190 | 1.986 | 0.142 |
|  | B (n=121) | 0.993 | 0.843 |  |  |
|  | C (n=131) | 0.980 | 0.048 |  |  |
| Day 2 | A (n=125) | 0.929 | ＜0.001 | 2.003 | 0.136 |
|  | B (n=121) | 0.985 | 0.205 |  |  |
|  | C (n=131) | 0.965 | 0.002 |  |  |
| Day 3 | A (n=125) | 0.945 | ＜0.001 | 61.764 | ＜0.001 |
|  | B (n=121) | 0.916 | ＜0.001 |  |  |
|  | C (n=131) | 0.910 | ＜0.001 |  |  |
| Day 4 | A (n=125) | 0.970 | 0.007 | 2.880 | 0.059 |
|  | B (n=121) | 0.986 | 0.239 |  |  |
|  | C (n=131) | 0.987 | 0.271 |  |  |
